# Treatment success rate and time to culture conversion under a prospective BPaL cohort study

**DOI:** 10.5588/ijtldopen.24.0524

**Published:** 2025-05-12

**Authors:** E. Burhan, J. Sugiharto, M. Soemarno, A. Juan, Y. Runtu, A. Yuvensia, R. Ramadhani, J. Sabono, A. Lailiyah, F. Fenni, M. Farikha, T. Pakasi, I. Pambudi, M. Mbenga, I. Koppelaar, V. Mirtskhulava, F. Wares, D. Jerene, J.K. Jung, J.S. Lee, S. Foraida, S. Juneja, M. Diachenko, A. Gebhard

**Affiliations:** ^1^ Department of Pulmonology and Respiratory Medicine, Faculty of Medicine, Universitas Indonesia, Jakarta, Indonesia;; ^2^ Persahabatan Hospital, Jakarta, Indonesia;; ^3^ Yayasan KNCV Indonesia, Jakarta, Indonesia;; ^4^ National Tuberculosis Programme, Ministry of Health of the Republic of Indonesia, Jakarta, Indonesia;; ^5^ Directorate of Mental Health, Ministry of Health of the Republic of Indonesia, Jakarta, Indonesia;; ^6^ Division of TB Elimination and Health Systems Innovations, KNCV Tuberculosis Foundation, The Hague, The Netherlands;; ^7^ Inpatient Care, Lelie Care Group, Rotterdam, The Netherlands;; ^8^ Faculty of Natural Sciences and Medicine, Ilia State University, Tbilisi, Georgia;; ^9^ Division of TB Elimination and Health Systems Innovations, KNCV Tuberculosis Foundation, Worthing, UK;; ^10^ Division of Global Health, International Tuberculosis Research Center, Changwon, Republic of Korea;; ^11^ Division of Microbiology, International Tuberculosis Research Center, Changwon, Republic of Korea;; ^12^ Research and Development, TB Alliance, New York, NY, USA;; ^13^ Market Access, TB Alliance, New York, NY, USA.

**Keywords:** cohort studies, treatment outcome, multidrug-resistant, culture conversion, BPaL, Indonesia

## Abstract

**BACKGROUND:**

In July 2022, Indonesia implemented the 6-month BPaL (bedaquiline, pretomanid, linezolid) regimen under operational research (OR) for selected drug-resistant tuberculosis patients. The study aimed to assess treatment success rate (TSR) and time to sputum culture conversion (TSCC).

**METHODS:**

A prospective cohort study in fifteen sites between July 2022 and March 2023 enrolled patients with rifampicin-resistant/multidrug-resistant TB with additional fluoroquinolone resistance or intolerance/failures of previous second-line TB treatment. TSR was descriptively analysed, and Kaplan-Meier and Cox proportional-hazards analyses were used to evaluate TSCC.

**RESULTS:**

A total of 87 patients were enrolled, 3 were withdrawn, and 84 completed treatment and had outcomes; 82 (97.6%) patients had successful treatment, 1 (1.2%) died, and 1 (1.2%) had failure. Overall, 61 (72.6%) patients had positive cultures at baseline, and favourable outcomes were included in the TSCC analysis; all 61 (100%) converted within the first 3 months (median 32 days of treatment, IQR 30.0–56.0). None of the six variables were statistically associated with conversion time.

**CONCLUSION:**

The Indonesian BPaL OR showed a highly promising TSR of 97.6%, with 100% sputum conversion within 3 months. The lack of observed statistical differences in the TSCC across variables shows that the BPaL treatment will be equally effective in all patient groups.

In 2023, Indonesia ranked second among five countries contributing 50% of the global gap between estimated TB incidence and the reported number of new TB diagnoses, and it was among 10 countries that accounted for 75% of the global gap in rifampicin-resistant/multidrug-resistant TB (RR-/MDR-TB) treatment enrolment.^1^

Challenges, including long treatment duration, high pill burden, and frequent side effects, further worsen the high drug-resistant TB (DR-TB) burden. The situation is more complicated for RR-/MDR-TB patients who have additional resistance to fluoroquinolones (FQs), i.e., pre-extensively DR-TB (pre-XDR-TB) cases, and who previously required long-term combination therapy lasting 18 to 24 months.^2,3^

A more effective treatment was needed. In 2020, the Nix-TB Study in South Africa investigated the effectiveness and safety of a novel DR-TB regimen containing bedaquiline (Bdq), pretomanid (Pa) and linezolid (Lzd) – the BPaL regimen – in extensively drug-resistant TB (XDR-TB) patients, using a previous WHO definition of XDR-TB of RR-/MDR-TB plus resistance to any FQ and any of the second line injectable agents, and MDR-TB patients with unresponsiveness/intolerance to their second-line regimen. Patients were treated for 26 weeks with the BPaL regimen, containing 1,200 mg daily Lzd (adjustable after 4 weeks) and 200 mg of Pa, with 400 mg of Bdq daily during the initial two weeks followed by 200 mg three days a week for the remaining 24 weeks. The regimen achieved a 92% treatment success rate (TSR) under modified intention-to-treat analysis.^4^

Later in 2020, WHO recommended the BPaL regimen for selected DR-TB patients under operational research (OR) for 6–9 months.^5^ Following this, Indonesia implemented the BPaL regimen in 2022 for selected DR-TB patients under OR conditions. The objectives of the OR were to evaluate the effectiveness and safety of the BPaL regimen. This article focuses on evaluating the effectiveness by analysing TSR and time to sputum culture conversion (TSCC) as key predictors of treatment outcomes in DR-TB patients.^6–8^ Common factors associated with TSCC include biological sex, age, type of TB resistance, presence of any chronic disease, baseline body mass index (BMI), and cavitary lesion.^9–12^ The TB-PRACTECAL study found that the percentage of patients with confirmed culture conversion who receive the 24-week all-oral treatment regimen for DR-TB, including the BPaL regimen, was higher than the 9-to-20-month standard of care regimen.^13^ However, there are presently no studies to identify predictors for addressing TSCC predictors among DR-TB patients undergoing BPaL treatment.

## METHODS

### Study design and participant

A prospective cohort study under OR conditions was conducted at 15 sites across four provinces in Indonesia with the highest DR-TB burden: the Special Capital Region of Jakarta, West Java, Central Java, and East Java. The OR protocol was adapted from KNCV TB Foundation’s Generic BPaL OR Protocol^14^ to meet the Indonesian setting, with a cohort of 100 patients planned.

Patient enrolment was from July 2022 to March 2023 and included RR-/MDR-TB patients who had FQ resistance (i.e. pre-XDR-TB) or were non-responsive/intolerant to prior second-line anti-TB treatments or had been in close household contact with an index pre-XDR-TB case. They also had no documented resistance to BPaL components based on phenotypic drug susceptibility testing (pDST) at baseline, were ≥18 years old, weighed ≥35 kg, gave informed consent, and adhered to the OR procedures.

Patients were excluded if they had an allergy or a history of serious adverse event (SAE) to any of the BPaL drugs; were pregnant or breastfeeding or planning to become pregnant during the OR period; had grade 3 or 4 peripheral neuropathy or grade 1–2 that was projected to worsen during treatment; refused contraception (reproductive-age women); had resistant to a BPaL drug; had meningitis, central nervous system, or osteomyelitis TB; were unable to ingest oral medication; or the Clinical Expert Team recommended an individualised regimen.

### Treatment regimen

Patients received 26 weeks of BPaL regimen, with 2 weeks loading dose of Bdq 400 mg, then 200 mg for 24 weeks following WHO recommendation,^15^ and 200 mg of pretomanid once daily. Sixty-three patients began with 1,200 mg of Lzd daily, with allowed dose reduction/discontinuation after 4 weeks of treatment due to adverse events (AEs). Following an updated protocol (per updated WHO guidance), 21 patients started with Lzd 600 mg daily, with allowed reduction/discontinuation after 9 weeks of treatment. If the sputum culture remained positive after 4 months, the patient received 3 additional months of BPaL (totalling 9 months) if clinically progressing.

### Treatment monitoring and evaluation

All eligible patients underwent baseline evaluation before enrolment and received routine treatment monitoring per National TB Programme (NTP) guidelines (Table 1).

**Table 1.** Baseline evaluation and treatment monitoring.

Evaluation’s type	Baseline	2 weeks after treatment initiation	Every month	End of treatment	6 months after treatment completion
Clinical evaluation					
Physical examination	√	√	√	√	√
Evaluation of psychosocial condition	√	√	√	√	√
Functional status	√				
Weight and BMI	√	√	√	√	√
Peripheral neuropathy screening	√	√	√	√	
Visual function screen	√	√	√	√	
Psychiatric screen	√	√			
Adverse reaction monitoring	√	√	√	√	
Treatment result consultation				√	√
Bacteriological evaluation					
Sputum smear	√		√	√	√
Sputum culture	√		√	√	√
Second-line LPA	√	To be repeated if AFB/culture is positive in the 4^th^ month, at the end of treatment, or post-treatment follow-up			
Phenotypic drug susceptibility test	√	To be repeated if AFB/culture is positive in the 4^th^ month, at the end of treatment, or post-treatment follow-up			
Laboratory, radiology and ECG evaluation					
Chest X-ray	√			√	
ECG	√	√	√	√	
Full blood count	√	√	√	√	
Liver function tests: ALT, AST, total bilirubin	√	√	√	√	
Serum electrolytes: Na, K, Ca, Mg	√		√		
Kidney function tests: (urea, creatinine)	√		√		
Blood sugar level (fasting and 2 h post-prandial)	√				
TSH/TSHs	√				
Pregnancy test	√				
HIV testing	√				

BMI = body mass index; LPA = line-probe assay; AFB = acid-fast bacille; ECG = electrocardiogram; ALT = alanine aminotransferase; AST = aspartate aminotransferase; Na = sodium; K = potassium; Ca = calcium; Mg = magnesium; TSH = thyroid-stimulating hormone.

Directly observed treatment was provided by a DR-TB nurse or patient supporter (either at the hospital or by video call) as per NTP guidelines. Active drug safety monitoring and management were conducted by healthcare workers at the respective healthcare facility.

### Variables

The study investigated the TSR, i.e., the proportion of enrolled patients with ‘cured’ or ‘treatment completed’ outcomes at the end of the BPaL treatment. The definitions followed the Indonesian BPaL OR protocol, based on the following WHO definitions and reporting framework for TB (updated December 2014):^16^

*Cured:* BPaL treatment completed without evidence of treatment failure and has at least two consecutive negative culture results in a row at least 30 days apart within the last 3 months of treatment.
*Treatment completed:* BPaL treatment completed, without evidence of treatment failure, but no negative culture results on at least two consecutive occasions in a row in the last 3 months of treatment. The final treatment outcome for patients with negative cultures at baseline examination was ‘Treatment completed’.
*Treatment failure:* Patients had treatment terminated or need for permanent regimen change of at least two anti-TB drugs because of lack of conversion or culture reverted after conversion to negative; treatment terminated early due to poor clinical or radiological response, or permanent discontinuation of Bdq or Pa or Lzd less than 9 weeks with 600 mg daily, due to AE.
*Died:* Patient dies for any reason during treatment.
*Lost to follow-up:* Patient who was interrupted for ≥ 2 consecutive months.
*Not evaluated:* Patient for whom no treatment outcome is assigned, including but not limited to patients withdrawn from the BPaL treatment due to protocol violation.

Successfully treated patients were offered a 6-month post-treatment follow-up evaluation. Smear and culture specimens were collected. Recurrent TB is defined as at least one positive culture with clinical and/or radiological findings suggestive of TB or two positive sputum cultures taken at least 30 days apart, regardless of clinical and/or radiological findings.

The study analysed six variables related to TSCC: biological sex, age, type of TB resistance, comorbidities, baseline BMI, and cavitary lesion(s). The TSCC was defined as the duration from DR-TB treatment initiation to the first of two consecutive negative cultures taken at least 30 days apart. Data from medical records, DR-TB programmatic forms, and TB information systems were recorded in the BPaL OR manual forms and entered into the Research Electronic Data Capture v9.7.5 (REDCap; Vanderbilt University, Nashville, TN, USA) system —a web-based application for OR data storage and analysis. The OR and site teams received training to standardise assessments and implement routine monitoring and data validation to address information bias.

### Statistical analysis

Patients’ characteristics and TSR were analysed descriptively. Kaplan-Meier survival and Cox proportional-hazards analysis were used to evaluate the hazard ratio (HR) for TSCC with significance set at *p* < 0.05, using STATA v14 (Stata Corp, College Station, TX, USA).^17^ Only patients with baseline positive sputum culture and favourable outcomes were included in the TSCC analysis.

### Ethical approval

Ethical approval was obtained at the national level from the Ethics Committee of the Faculty of Medicine, University of Indonesia–Cipto Mangunkusumo Hospital, Jakarta, Indonesia (approval letter: KET-258/UN2.F1/ETIK/PPM.00.02/2022, 14 March 2022), and by the ethical committee at the OR sites.

## RESULTS

### Patients’ characteristics

**Figure 1.**
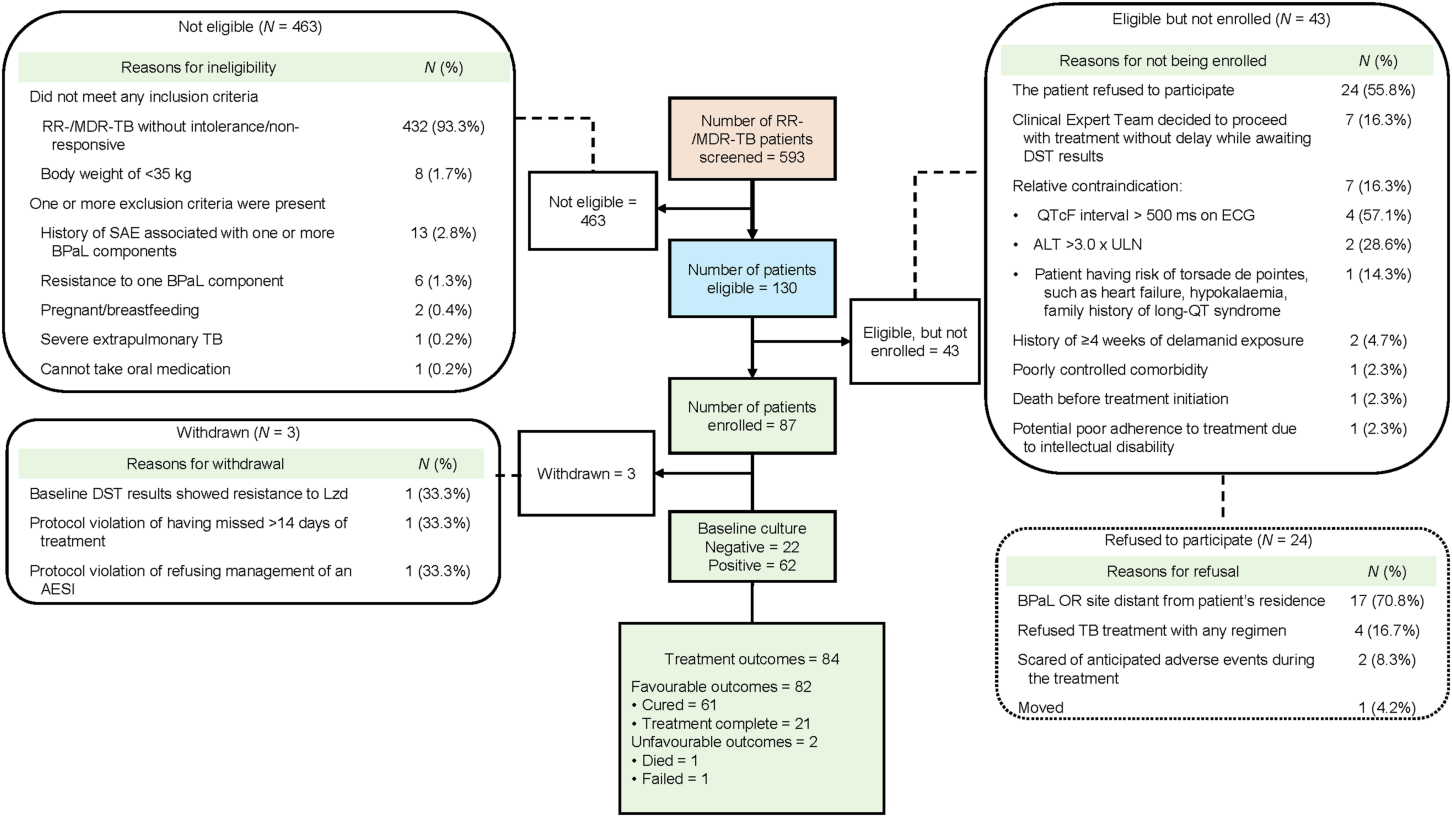
DR-TB patients of OR BPaL in Indonesia. RR-/MDR-TB = Rifampicin-resistant/multidrug-resistant TB; SAE = serious adverse event; BPaL = bedaquiline, pretomanid, linezolid; DST = drug susceptibility testing; Lzd = linezolid; AESI = adverse event of special interest; ECG = electrocardiogram; ALT = alanine aminotransferase; ULN = upper limit of normal; OR = operational research.

A total of 593 RR-/MDR-TB patients were screened, with 87 patients (67.0% of eligible) enrolled across the 15 sites. Three patients were withdrawn, including one with Lzd resistance based on baseline pDST results available 2 months after enrolment (received 61 doses of BPaL). Another patient missed 47 doses of the BPaL (received 166 doses of BPaL) due to socioeconomic problems and, as per the OR protocol, was categorised as a ‘protocol violation’. Both patients were switched to a more extended treatment regimen. Additionally, one patient who refused AE management and any further treatment received 74 doses of BPaL. In total, 84 patients completed BPaL treatment (Figure 1). About 54.8% were male, and 73.8% were working-age adults (18–54 years) (Table 2).

**Table 2.** Demographic and clinical characteristics of 84 patients with evaluated treatment outcomes.

Variable	*n* (%)
Total	84 (100)
Biological sex	
Male	46 (54.8)
Female	38 (45.2)
Age, years	
<35	25 (29.8)
35–44	16 (19.0)
45–54	21 (25.0)
≥55	22 (26.2)
Type of TB resistance	
RR	19 (22.6)
MDR	21 (25.0)
Pre-XDR	44 (52.4)
Comorbidity*	
No	43 (51.2)
Yes	41 (48.8)
Type 2 diabetes mellitus	31 (36.9)
Hypertension	9 (10.7)
Mental disorder	6 (7.1)
Kidney disease	3 (3.6)
Cardiovascular disease	2 (2.4)
Hepatitis B	1 (1.2)
HIV	1 (1.2)
Other	2 (2.4)
Baseline BMI, kg/m^2^	
Underweight (<18.5)	37 (44.0)
Normal (18.5–22.9)	36 (42.9)
Overweight (≥23.0)	11 (13.1)
Cavitary lesion(s)	
No	55 (65.5)
Yes	29 (34.5)

* A single patient may present with multiple comorbidities.

RR = rifampicin-resistant; MDR = multidrug-resistant; XDR = extensively drug-resistant; BMI = body mass index.

### Treatment outcomes

Out of the 84 patients, 82 (97.6%) had treatment success (61 cured, 21 completed treatment), 1 (1.2%) died, and 1 (1.2%) had treatment failure. The baseline pDST result for patients with treatment failure showed susceptibility to isoniazid, moxifloxacin, Bdq and Lzd and resistance to levofloxacin. DST was not done for rifampicin, kanamycin, amikacin, capreomycin and Pa. The patient died 10 weeks after completing their 6 months of BPaL treatment caused by sepsis due to moderate-severe Pneumocystis pneumonia (PCP). Unfortunately, a pDST test after BPaL treatment completion was not requested by the site clinical expert team.

### Time to sputum culture conversion

**Figure 2.**
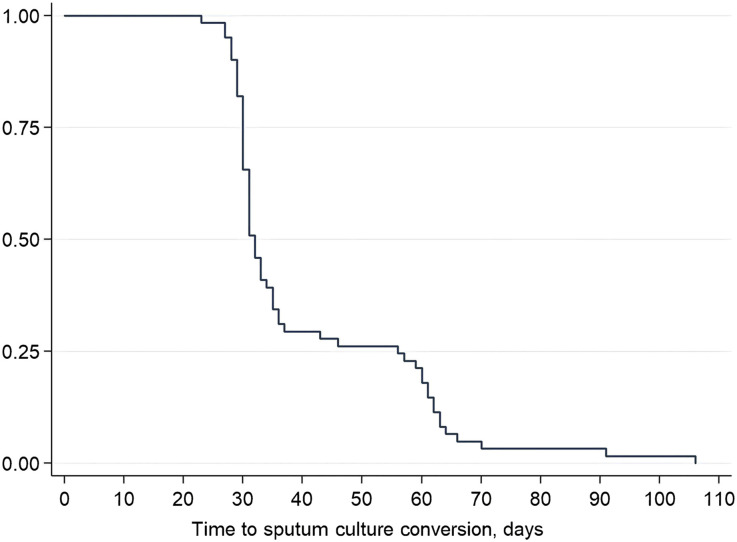
Time to sputum culture conversion.

Among the 84 patients with end-of-treatment outcomes, 61 (72,6%) had positive baseline cultures and achieved favourable outcomes. These 61 patients were included in the analysis. Of them, 52 (85.2%) had a complete series of monthly sputum examinations, while the remaining patients missed at least one. All 61 patients (100%) culture converted within the first 3 months of BPaL treatment, with 46 (75.4%) converting within the first month of treatment (Figure 2). The bivariate analysis of the six independent variables indicated no association with conversion time (Table 3).

**Table 3.** The analysis of factors related to sputum culture conversion time using Cox proportional hazards.

Variable	Frequency	Time to culture conversion	Cox proportional hazards	
	*n* (%)	Median [IQR]	HR (95% CI)	*P*-value
Total	61 (100.0)	32 [30–56]		
Biological sex				
Male	31 (50.8)	32 [30–59]	Reference	—
Female	30 (49.2)	31.5 [30–36]	1.05 (0.63–1.76)	0.84
Age, years*				
<35	16 (26.2)	32.5 [30.5–48.5]	Reference	—
35–44	12 (19.7)	31 [29.5–33.5]	1.48 (0.69–3.17)	0.31
45–54	18 (29.5)	31.5 [30.5–57]	0.98 (0.49–1.96)	0.96
≥55	15 (24.6)	31 [30–59]	0.92 (0.45–1.89)	0.82
Type of TB resistance				
RR	14 (23.0)	30 [29–46]	1.03 (0.44–2.42)	0.95
MDR	9 (14.8)	33 [31–35]	Reference	—
Pre-XDR	38 (62.3)	32 [30–60]	0.76 (0.36–1.61)	0.48
Comorbidity				
No	30 (49.2)	32.5 [31–61]	Reference	—
Yes	31 (50.8)	31 [30–46]	1.14 (0.68–1.91)	0.61
Baseline BMI, kg/m^2^^†^				
Underweight (<18.5)	25 (41.0)	31 [30–35]	Reference	—
Normal (18.5–22.9)	27 (44.3)	35 [30–60]	0.88 (0.51–1.54)	0.66
Overweight (≥23.0)	9 (14.8)	33 [31–37]	1.08 (0.50–2.35)	0.84
Cavitary lesion				
No	38 (62.3)	34 [30–60]	Reference	—
Yes	23 (37.7)	31 [29–34]	1.29 (0.76–2.18)	0.35

* The age group classification refers to the age group used in the WHO Global TB Report 2022, with adjustments on the lower and upper limits due to the data distribution.

† Categories refer to the WHO 2000 Western Pacific Region for Obesity.

IQR = interquartile range; HR = hazard ratio; CI = confidence interval; RR = rifampicin-resistant; MDR = multidrug-resistant; XDR = extensively drug-resistant; BMI = body mass index.

Of 82 eligible patients (i.e. those cured and those treatment completed), 69 attended their 6-month post-treatment follow-up assessment, with no cases of recurrent disease observed. Sixty-eight patients were culture-negative, and one asymptomatic patient had no sputum examination.

## DISCUSSION

This study found that the BPaL regimen showed high effectiveness, with a 97.6% treatment success rate, consistent with Nix-TB (92%)^4^ and TB-PRACTECAL (77%)^13^ studies. A similar study in Thailand reported a 90% success rate among DR-TB patients receiving BPaL treatment.^18^ Compared to Indonesia’s current DR-TB treatment success rate of 56% for the 2021 cohort,^19^ the BPaL regimen can improve treatment success if implemented extensively.

Of 84 patients, 61 had positive sputum culture at baseline with a favourable treatment outcome, with 75.4% converting within the first month and 100% within the first 3 months (median 32 days) of BPaL treatment. However, a contrast can be drawn with studies investigating TSCC in DR-TB patients receiving other available DR-TB treatment regimens, showing a median time to conversion of >60 days.^7,8,10^

In our study, two patients had an unfavourable treatment outcome, which was discussed with the project lead, the medical lead and the LIFT-TB project Scientific Committee. One patient died in the 10^th^ week of BPaL treatment due to poor management of pre-existing comorbidity (i.e. chronic heart failure) after refusing hospitalisation. Another patient, who initially converted within the first month, subsequently reverted at the end of BPaL treatment and was reported as a ‘treatment failure’. While adherence to BPaL was good, the patient’s HIV co-infection was poorly managed, with very poor adherence to their antiretroviral (ARV) treatment. As both HIV and other non-HIV comorbidities contribute to poor treatment outcomes in TB patients, the reason for the two patients who had ‘unfavourable outcomes’ was decided not directly due to the BPaL regimen. Comprehensive comorbidity management, including strict adherence to ARV therapy for HIV patients, is paramount in ensuring a favourable treatment outcome.^20–23^

This study found no correlation between biological sex, age, type of TB resistance, comorbidities, baseline BMI, and cavitary lesion to conversion time. This result is in line with several studies with non-BPaL regimens. A study in China (24–27-month treatment) found no significant association between age or biological sex and the time to sputum culture conversion.^7^ Another similar study in India also showed that age, biological sex, type of TB resistance, and cavitary lesion were not associated with TSCC.^24^ A study in Ethiopia (8–12 months of treatment) also found no significant association between age, biological sex, type resistance, BMI and comorbidity with TSCC.^8^ Based on the lack of observed statistical differences in the TSCC across these variables, we conclude that the BPaL treatment will be equally effective in all patient groups enrolled in the OR.

As the TSCC is currently considered a useful prognostic tool to predict end-of-treatment outcomes, anticipating predictors of sputum culture conversion could contribute to mitigating any potential non-response to treatment.

The study encountered several challenges: 43 of the 130 eligible patients did not enrol (Figure 1). Hence, although the planned cohort was for 100 patients in Indonesia, the number enrolled was 87, limiting the level of detail in the analysis. Furthermore, the method used to select the study locations, specifically focusing on DR-TB hospitals with a high burden of DR-TB cases and with three patients excluded from the outcome analyses, may have introduced a selection bias as the OR patient cohort may not fully represent the characteristics of the broader DR-TB services or patient populations across Indonesia. Additionally, AE and other potential predictors, including smoking patterns, chronic disease severity, etc., were not included in the analysis and might have influenced the results.

## CONCLUSION

Under the BPaL OR in Indonesia, selected DR-TB patients achieved a promising 97.6% success rate, suggesting BPaL’s potential to enhance DR-TB treatment outcomes. Unfavourable outcomes were observed in two patients with poorly managed comorbidities. Comprehensive patient care and multi-specialty care may improve the management of TB patients with pre-existing comorbidities. Of 61 patients, 75.4% culture-converted within the first month and 100% within 3 months. No significant association was found between demographic or clinical factors and conversion time. Close monitoring treatment and anticipating conversion time predictors are crucial for timely decision-making and mitigating potential non-response.
